# Advantages of IoT-Based Geotechnical Monitoring Systems Integrating Automatic Procedures for Data Acquisition and Elaboration

**DOI:** 10.3390/s21062249

**Published:** 2021-03-23

**Authors:** Andrea Carri, Alessandro Valletta, Edoardo Cavalca, Roberto Savi, Andrea Segalini

**Affiliations:** 1 ASE—Advanced Slope Engineering S.R.L., Via Robert Koch 53/a, Fraz. Pilastrello, 43123 Parma, Italy; andrea.carri@aseltd.eu (A.C.); roberto.savi@aseltd.eu (R.S.); 2 Department of Engineering and Architecture, University of Parma, Parco Area delle Scienze 181/a, 43124 Parma, Italy; edoardo.cavalca@studenti.unipr.it (E.C.); andrea.segalini@unipr.it (A.S.)

**Keywords:** landslide, monitoring, Internet of things, real time, early warning systems

## Abstract

Monitoring instrumentation plays a major role in the study of natural phenomena and analysis for risk prevention purposes, especially when facing the management of critical events. Within the geotechnical field, data collection has traditionally been performed with a manual approach characterized by time-expensive on-site investigations and monitoring devices activated by an operator. Due to these reasons, innovative instruments have been developed in recent years in order to provide a complete and more efficient system thanks to technological improvements. This paper aims to illustrate the advantages deriving from the application of a monitoring approach, named Internet of natural hazards, relying on the Internet of things principles applied to monitoring technologies. One of the main features of the system is the ability of automatic tools to acquire and elaborate data independently, which has led to the development of dedicated software and web-based visualization platforms for faster, more efficient and accessible data management. Additionally, automatic procedures play a key role in the implementation of early warning systems with a near-real-time approach, providing a valuable tool to the decision-makers and authorities responsible for emergency management. Moreover, the possibility of recording a large number of different parameters and physical quantities with high sampling frequency allows to perform meaningful statistical analyses and identify cause–effect relationships. A series of examples deriving from different case studies are reported in this paper in order to present the practical implications of the IoNH approach application to geotechnical monitoring.

## 1. Introduction

Nowadays, monitoring systems play a key role in the study and description of natural phenomena, together with the analyses for what concerns risk prevention. This allows obtaining useful information for continuous and optimal management of infrastructures, landslides and critical events in general [1]. The geotechnical field has traditionally been characterized by a manual approach regarding both data collection and on-site investigations. This methodology can be quite complex and time-consuming [2], particularly for environments and sites difficult to access directly, especially during hazardous events. Due to these reasons, innovative tools integrating different sensors have been developed in the last two decades thanks to technological improvements [3]. The main goal is to produce a complete and efficient system featuring automatic procedures and characterized by higher accuracy, reliability and durability compared to traditional devices.

One of the major advancements introduced by innovative instrumentations is the possibility to overcome the so-called “manual philosophy”, thanks to the integration of Internet of things (IoT) technologies in the design process of automated monitoring systems [4,5,6,7]. When it comes to landslide monitoring, the integration of IoT-based devices results in a multilayered structure, where each component has a key role in guaranteeing the system functionality and efficiency [8,9,10]. While the architecture could vary from one case study to another, it is possible to identify some essential components for IoT-based monitoring systems:

- The perception layer, consisting of a network of connected sensors responsible for collecting the data and transmit them to the elaboration center;
- The data layer, which entails dataset storage and elaboration. This is an essential component in an IoT-based system since traditional approaches are usually unable to manage the amount of data generated by an automatic system;
- The presentation layer, where monitoring outcomes are represented and made available for the end-user through dedicated mobile applications or web-based services.

This approach has found successful applications in the geotechnical monitoring field, with the integration of both traditional and innovative sensors for monitoring and early warning purposes. Some examples include the multi-device wireless monitoring of a sloped surface [11], as well as sensors columns for the detection of the formation of a slip surface [12], and optical instrumentation to acquire images and detect potentially critical events [13]. In all these configurations, the main advantage relies on the possibility of achieving a real-time or near-real-time monitoring approach through the integration of automated processes and multiparameter tools. Moreover, IoT-based monitoring systems have been employed in underground excavation works, specifically for early warning purposes. For example, multiparameter approaches have been introduced in subway construction sites, where traditional EWS based on single-sensor data and simple models are not suitable for such complex frameworks [14,15,16]. In fact, more recent systems are able to acquire information from multiple sensors thanks to the integration of wireless networks to connect every device installed on-site [16,17,18].

The implementation of IoT-based procedures is the core component of the Internet of natural hazards (IoNH) approach [19]. IoNH represents a design concept where different system components can interact and exchange data thanks to Internet connectivity. This is achieved by exploiting a bidirectional remote control of different sensors and control units (CU) placed in a specific monitoring site, taking advantage of a cloud-based database (DB), software and representation platform. Equipped with appropriate technology, network-connected devices can be remotely controlled and accessed, thus creating an integrated and automated monitoring system with improved efficiency and performance.

The ability of IoT tools to automatically acquire and elaborate monitoring data has laid the basis for the development of dedicated self-learning or machine-learning automatic software, able to analyze the dataset and apply self-check controls in order to provide a preliminary validation of raw data, excluding outliers and sensors errors. The statistical validation is possible thanks to the high sampling rate available with modern technologies (e.g., CU and DB services). IoNH also relies on the possibility to record a large number of different parameters and physical quantities, obtaining a more accurate description of the ongoing phenomenon. This aspect permits to definition cause–effect relationships or failure predictions, sometimes only based on numerical data. Moreover, an automatic procedure would permit monitoring even rapid and impulsive events that manually operated tools would not be able to identify due to their lower sampling frequency.

The management process requires the design and application of web-based representation platforms, making the monitoring results easily and quickly accessible for further analyses and interpretation [20]. This aspect should not be underestimated since the timely evaluation of sensor outcomes leads to effective intervention strategies, e.g., people evacuation from an area at risk or road closure before the occurrence of a critical event. Additionally, automatic systems based on solid calculation algorithms are a key component in the design of landslide early warning systems (EWS), which need to be able to detect landslide activities as soon as possible [21].

This paper will focus on the features and applications of the IoNH system, which is intended to provide a new methodology to overcome the traditional approach that characterizes the geotechnical monitoring field. Particular attention will be dedicated to the different advantages resulting from the integration of these IoT-based components into automatic monitoring systems. Moreover, a series of examples taken from different case studies are reported in order to underline the methodology value in different aspects of the monitoring activity, e.g., power supply control, data elaboration procedures, cause–effect correlations, etc. Additionally, the following sections report a description of the IoNH structure and components, also exploring some details regarding the algorithms involved in the elaboration process.

## 2. Materials and Methods

**Figure 1:**
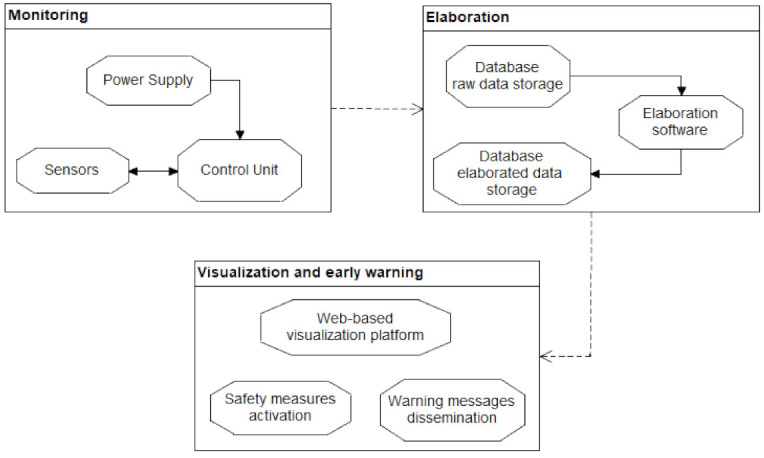
Internet of natural hazards (IoNH) approach applied to modular underground monitoring system (MUMS) system, with data collection, transmission, database storage, automatic elaboration, results representation, alarms activation.

The IoNH approach is based on the experience acquired along 7 years of automatic geotechnical monitoring through a modular underground monitoring system (MUMS), from on-site sensors acquisition to results representation. MUMS was designed as an automatic inclinometer composed of several nodes (links) located at custom distances along a single chain, linked by an aramid fiber and a quadrupole electrical cable. The array functionality is automated, collecting multiparametric data through a remotely controlled datalogger and RS485 protocol [22]. Since its first application as a device to monitor landslide displacements, MUMS technology has been developed in order to adapt to different geotechnical scenarios, and it is nowadays the core of several tools intended for other applications (e.g., underground excavations, civil and geotechnical structures, rockfall and debris flow barriers, and geothermal plants). The IoNH working principle is common to all these applications and is summarized in Figure 1.

### 2.1. Control Unit

CU queries sensors at the specified sample period and applies the first filter on the raw dataset, performing 64 readings and saving the mean value in an external SD card. Moreover, the datalogger is able to record any kind of traditional sensors featuring an analog output signal. This particularly useful feature permits the management of complex and diversified monitoring systems with a single logger, DB, software and web platform, with relevant advantages in terms of data management and visualization. The CU power supply comes from a lead battery recharged through a solar panel or, if possible, directly connected to the electrical line. Both these configurations are intended to provide a reliable power source, which is an absolute necessity for any acquisition system [23]. Moreover, the elaboration software analyses the voltage and automatically sends warning messages, while the battery level is available in the web-platform. This aspect represents a significant upgrade with respect to traditional automated, non-remotely controlled devices since the inability to verify the control unit functionality could lead to potential losses of monitoring information.

### 2.2. Sensors

As anticipated in previous paragraphs, one of the main features of the IoNH approach is the possibility to integrate a wide selection of different sensors. This characteristic is intended to provide a flexible and customizable system, able to adapt to different contexts according to the relevant parameters to be monitored. The most common configuration, designed to measures displacements in landslides and underground constructions, relies on microelectromechanical system (MEMS) sensors, composed of an accelerometer, a magnetometer, and a thermometer. In those cases where a higher resolution is needed (e.g., monitoring of structures and buildings), it is possible to integrate an electrolytic tilt sensor. It is worth mentioning that MEMS have a 360° measuring range, while electrolytic cells feature a full-scale value of ±25°. Additionally, the structure of MUMS-based arrays permits the exploitation of different sensor typologies in the same array, such as piezometers to detect water level variations [24] or high-accuracy thermometers to have a detailed description of the underground temperature at different depths [25]. Finally, IoNH allows the acquisition and elaboration of monitoring data sampled by traditional instrumentation featuring an analog output signal (e.g., extensometers, crack meters, load cells, etc.), which can also be integrated into a monitoring system, including digital output tools. This particular configuration can usually be observed when dealing with tunnels and underground excavations, where the monitoring activity involves a large number of different elements of the structure and the surrounding environment [26].

### 2.3. Data Transmission and Storage

The data transmission generally relies on 4G, 3G or GPRS lines with a UMTS router, while in some cases, it is also possible to use fiber optics leading to a local elaboration center. Raw data (electrical signals) are stored in a cloud DB and always available for a back analysis or subsequent elaborations by exploiting new algorithms or statistical filters. In the case of monitoring systems based on the IoNH structure, the elaboration process takes into account several variables, resulting in a quite complex and potentially expensive procedure from a computational point of view. For this reason, the system is designed to transmit raw data and calculate the results at the elaboration center or, alternatively, run on a specific elaboration center on-site.

### 2.4. Elaboration and Self-Check Controls

The elaboration algorithm, specifically designed for each MUMS-based application, converts electrical signals into physical units, removes spike noises and accidental errors, and calculates the final results. Self-check data controls are a fundamental aspect when dealing with the management of automated tools. In particular, MUMS devices exploit a multilevel automated control procedure implemented in the elaboration algorithm, relying on the verification of specific physical entities listed below [27]:

- Recognition of spike noise;
- Coherence of gravity acceleration vector;
- Variability of gravity acceleration vector;
- Coherence of magnetic field vector;
- VVariability of magnetic field vector;
- Coherence of node temperature;
- Running average;
- Recognition of instrumental noise;
- Recognition of not-operating sensors.

#### 2.4.1. Recognition of Spike Noise (I)

Spike noise (or impulse noise) is a sporadic and impulsive electrical disturb, related to sharp and sudden disorder in the signal, often related to external factors, which heavily contaminate the acquired data [28]. The identification of spike noise exploits statistical tools over a specific dataset. For example, the MUMS algorithm analyses the statistical dispersion evaluating the variability of a univariate sample of quantitative data with the scaled median absolute deviation (MAD). MAD represents a measure of the statistical dispersion, and it is defined as the median of the absolute deviations from the data’s median [29], as in Equation (1). The removal of accelerometer spike noise is particularly important in some applications, such as tunneling and underground excavations in general, where the vibrations induced by works could highly influence the single data-point, resulting in a well-identifiable outlier [27]:

(1) $$MAD=c*median\left(\left|X_{i}-median\left(X\right)\right|\right)$$

(2) $$c=\frac{-1}{\sqrt{2}*erfc^{-1}\left(\frac{3}{2}\right)}$$

(3) $$erfc\left(x\right)=\frac{2}{\sqrt{\pi }\int_{x}^{\infty }e^{-t^{2}}dt}=1-erf\left(x\right)$$

(4) $$erf\left(x\right)=\frac{2}{\sqrt{\pi }}\int_{0}^{x}e^{-t^{2}}dt$$

where:

- $X_{i}$ is the data to be evaluated;
- $erfc\left(x\right)$ is the complementary error function;
- $erf\left(x\right)$ is the error function [30].

The recognition of spike noises shows high performances if the dataset is centered [27]. For this reason, when dealing with real-time data transmission, the calculation of the last part of the data sample is not completely validated until its time window is fully centered. The immediate consequence is a time delay for the validation of the results, strictly related to the number of points considered, the sample period, and the data transmission frequency (as in Equation (5)):

(5) $$out_{i}=f\left(t_{sp},t_{ft},d_{w}\right)$$

where:

- $out_{i}$ is the identification process of outlier *i*;
- $t_{sp}$ is the sampling period;
- $t_{ft}$ is the frequency of data transmission;
- $d_{w}$ represents the dimension of the dataset window.

$t_{sp}$, $t_{ft}$ and $d_{w}$ parameters should be defined accordingly to the monitoring needs. The sampling period is related to the most probable phenomenon evolution, with a higher sampling rate linked to a rapid displacement (Equation (6)). Thanks to the improved connection between elements included in IoNH systems, it is possible to update the $t_{sp}$ the value during the monitoring process, with an automated or a manual procedure performed by remote. Moreover, $t_{sp}$ is strongly dependent on the dataset window dimension (related to the robustness of the statistical analysis) and electrical power ($e_{p}$), as in Equation (6):

(6) $$t_{sp}=f\left(v,d_{w},\;e_{p}\right)$$

The frequency of data transmission is an important parameter because it determines the data elaboration and consequently the time period ($t_{r}$) related to the providing of final results (Equation (7)). This parameter should coincide with the sampling period if the monitoring has early warning system purposes in order to have timely information regarding the phenomenon evolution. Therefore, this process usually requires more electrical power supply than the sampling procedure, especially for remote areas where the 3G phone line is not well covered. These parameters should be considered at the monitoring design stage in order to have a proper power supply (where it is possible):

(7) $$t_{ft}=f\left(e_{p},v,d_{w},\;t_{r}\right)$$

Finally, regarding the dimension of the dataset window ($d_{w}$), it should be considered that a reduced dataset validates the raw data in a shorter time but could not be statistically valid [27]. A large dataset strengthens the statistical processes and recognition of instrumental and accidental noise, but it comes with an increase in validation and calculation time. Therefore, a monitoring system involving these procedures should be described as a “near-real-time” application instead of “real time”, due to all processes needed for data acquisition and elaboration [31].

#### 2.4.2. Coherence and Variability of Gravity Acceleration and Magnetic Field Vector (II, III, IV and V)

MUMS system usually relies on 3D MEMS sensors featuring three main components, namely an accelerometer, a magnetometer, and a thermometer. Thanks to its three-dimensional functioning principle, it is possible to reconstruct the magnetic field vector, represented by gravity acceleration *g*. Moreover, the static nature of these measures allows controlling the coherence and variation of instrumental data at every step. The coherence, as in Equation (8), identifies sensors with relevant malfunctions that would undermine their calibration. The variation (Equation (9)) could be related to instrumental noise that characterizes every electronic device, and it is eligible under a certain threshold (${∆}_{g}$) that should be defined accordingly to sensor technical features. Automated filters identify uncorrected values and adjust the related results, giving a preliminary software validation to the dataset. Since they exploit the resultant vector in a 3D space, previously mentioned controls are not possible using a 2D MEMS:

(8) $$\frac{\sqrt{a_{x}^{2}+a_{y}^{2}+a_{z}^{2}}}{g}=1$$

(9) $$\left|1-\frac{\sqrt{a_{x}^{2}+a_{y}^{2}+a_{z}^{2}}}{g}\right|\geq {∆}_{g}$$

#### 2.4.3. Running Average (VII)

Running average process is complementary to the recognition of spike noise, and it is applied to identify and reduce instrumental noise by eliminating fluctuations smoothing the analyzed dataset [32]. The operation is fundamental when the expected displacements have the same order of magnitude as instrumental sensitivity and repeatability [27]. Together with the mentioned advantages in terms of the improved sensor performances, this operation should be avoided when rapid and impulsive displacements are expected. In this specific case, the acceleration phase is highly anticipated, consistently reducing the displacement rate and the correct phenomenon identification. A possible solution could be obtained by increasing the sampling period (with consequently incremented electrical consumption).

### 2.5. Web Platform and Alarms

Outcomes of interest are represented through a dynamic and intuitive web platform with secure, controlled access. This passage is of fundamental importance since the installation of a huge number of instruments with multiparametric data requires adequate data management (following this approach, the design process should aim to simplify and optimize the visualization and interpretation of results, leaving time-expensive operations, such as organization and representation processes to automated systems.

Web-based environments provide several advantages in terms of accessibility and performances, thanks to the implementation of a remote-user-friendly interface, which allows interacting with a continuously updated system [33]. Moreover, the integration of automatic processes gives the possibility to customize the platform, allowing the users to interact easily with monitoring data according to specific needs (e.g., selecting a specific time interval, downloading data plots in several formats, etc.) Finally, this approach permits to configure automated reports, which periodically summarize the site conditions. The main objective is to highlight specific events like the increasing of displacement rates or pore pressure, together with other conditions diverging from the historical data trends. The management of a large number of multiparameter data with high sampling frequencies sometimes permits the establishment of significant cause effects relationships, with direct correlations between rainfalls, water levels and displacements over specific periods.

A smart IoNH management system should automatically change CU configuration parameters, like sampling period, data transmission frequency, etc., according to monitoring outcomes. In this way, it is possible to overcome some issues related to the elaboration process, e.g., the disadvantages related to the application of running average, as mentioned before. In addition, an increase in the sampling period highly improves the performances of failure forecasting models for early warning applications [34]. On the other hand, if the battery voltage is reaching critical levels, the management system should automatically reduce sampling and transmission frequency.

The activation of alert or alarm procedures is another key element that composes an IoNH system. Traditional methods involve the definition of thresholds and, consequently, the activation of predefined procedures at their overcome. This philosophy requires the definition of the landslide mechanical model or, in general, the monitored phenomenon [35]. The approach could be characterized by multilevel or single-level solutions, involving a very clear distinction without any intermediate level, and its effectiveness depends on several critical factors. First, it is necessary to carry on-site investigations in order to create the landslide geological and geotechnical model. This should be followed by a mechanical numerical model that tries to reproduce the monitoring outcomes [36] starting from materials characteristics. In general, the procedure requires several months of monitoring data and the employment of well-prepared technicians. This leads to significant time and cost requirements that should be taken into account since their availability should not be taken for granted. Other methods exploit only monitoring data without taking into account any mechanical description of the phenomenon. These approaches use failure forecasting models, like the Inverse Velocity Method [37,38], in order to define thresholds or activate warning procedures related only to displacement rate and acceleration data. These approaches involve some hypotheses and assumptions in order to be applied and are not able to guarantee a completely accurate representation of every possible phenomenon. Therefore, it is usually recommended not to apply these models in isolation to avoid possible misinterpretations [39].

**Figure 2:**
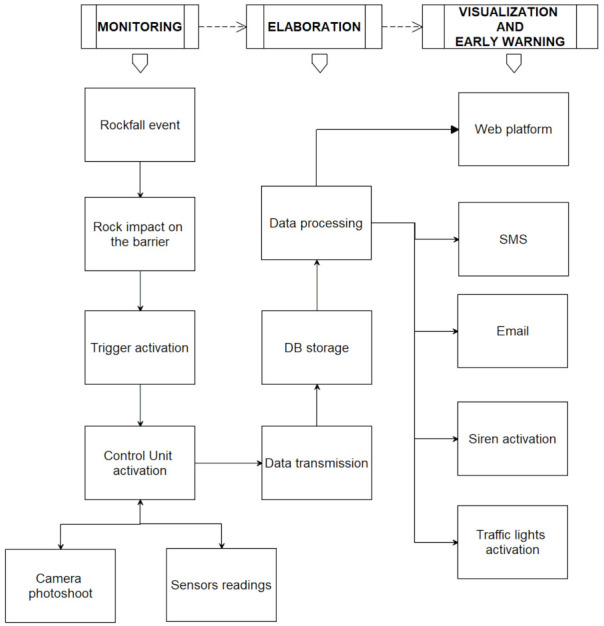
IoNH approach applied to a rockfall event and its impact on protection barriers, followed by trigger activation, data collection and transmission, database storage, data processing, and activation of warning procedures.

Finally, another application takes advantage of trigger or shock sensors. These instruments can be mechanical or electronic devices and are designed to detect impulsive phenomena like the impact of a mass against a barrier or a debris flow occurrence [40,41,42]. Depending on the device, these sensors usually consist of a steel cable, a dynamic MEMS or a geophone. In the first case, the cable could be wrapped up in the barrier, activating a trigger signal when the rope is pulled. A dynamic MEMS works as a shock sensor and issues warnings at the overcoming of a predetermined acceleration threshold. The geophone has a very similar working principle as a dynamic MEMS sensor and records vibrations or seismic waves that could anticipate the arrival of a critical event. The trigger activation could directly lead to alert procedures, or it could be integrated within cross-check controls. These processes analyze other relevant physical entities like steel posts tilt or load exerted on ropes. As an example, an application of IoNH to rockfall barriers monitoring is represented in Figure 2. A rock mass hits the barrier, pulling a mechanical device. This one triggers the CU, which starts to read every sensor connected with a local smart mesh network and activates a photoshoot captured with a small camera. Information is sent to the elaboration center, where a self-learning software analyses the sensor results. This operation could be tackled in different ways by using absolute thresholds, relative thresholds or by comparing the last data received with the previous data trend. According to predetermined conditions or warning levels, the procedure could send emails (with the photoshoot attached) and/or SMS to the authorities in charge while activating sirens and/or traffic lights placed on the site access roads at the same time.

## 3. Results and Discussion

**Figure 3:**
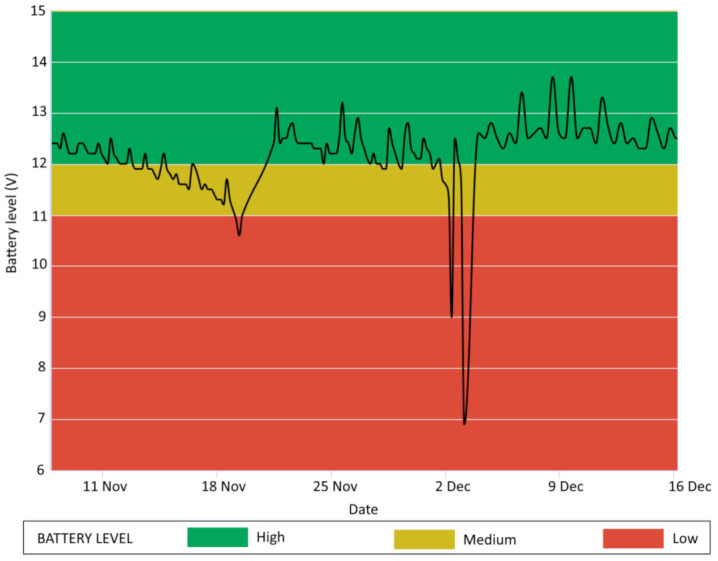
Battery voltage monitoring over time, with three different charge levels.

As discussed above, a major task of an IoNH system is the monitoring of CU battery voltage. Due to their nature, rechargeable lead–acid batteries lose their functionality when the level goes down a critical value, causing a potential loss of data. For this reason, the automated software is able to send warning messages to monitoring responsible for the occurrence of critical situations in order to take timely actions and restore the system functionality. Figure 3 presents an example of this procedure, displaying a case study where the battery voltage monitoring allowed to identify relevant reductions of the charge level. In particular, the system involved an array of 23 high-resolution thermometers for the monitoring of soil temperature at different depths. The instrumentation was set on a 10-min sampling frequency, while a lead–acid battery connected to a solar panel provided the power supply to the monitoring system. After the tool installation, a prolonged period of bad weather in the area hindered the ability of the solar panel to properly recharge the battery. This event, coupled with the particularly high sampling frequency, generated a decreasing trend of energy supply. Thanks to alert messages issued in correspondence to these events, it was possible to address the problem with appropriate on-site operations, thus avoiding more serious malfunctions. In particular, after the substitution of the battery and the upgrading of the solar panel with a more powerful one, at the beginning of December, the system reached a stable configuration and was able to continue regularly the monitoring activity.

**Figure 4:**
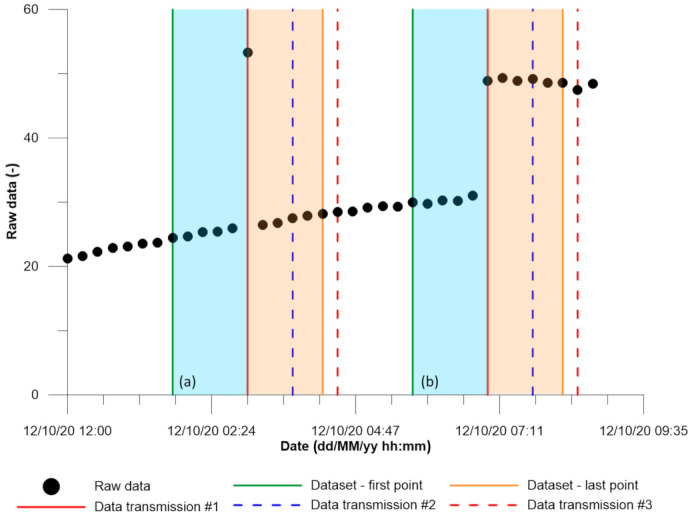
Raw data analysis, focusing on a spike event (**a**) and an actual displacement (**b**), was performed by using an 11-element data window centered on the continuous red line points, ranging between the green line and the orange line. Data transmissions are represented by red, dashed blue and dashed red lines [27].

The removal of outliers and spike noise is a relevant topic when dealing with EWS, even if the process causes a time-delay related to the complete dataset validation. For example, Figure 4 reports a monitoring dataset characterized by a sample period of 10 min, a statistical analysis that considers 11 elements and a data transmission frequency to the elaboration center of 30 min [27]. Because of the delay between data acquisition and elaboration, this specific case study qualifies as a “near-real-time” monitoring setup, according to the observations reported in the previous section. When the information to be evaluated reaches the database, the calculation process could apply only a left-weighted de-spiking process, using a dataset, which starts 5 points before (green lines in Figure 4). If the data to be evaluated does not follow the previous trend, the algorithm could potentially identify two scenarios: the occurrence of a spike (Figure 4, case a) or the beginning of a new movement trend (Figure 4, case b). The software must perform a preliminary real-time analysis that interprets one of the two mentioned cases, accordingly to the deviation from the median. This information has a 50% reliability [27]. After 30 min a new data transmission takes place (blue dashed line), adding 3 new information to the dataset. The new elaboration takes advantage of these elements, and the algorithm identifies the occurrence of a spike (Figure 4, case a) or a displacement (Figure 4, case b). The net result has higher confidence (80%) than the previous one, keeping a small uncertainty due to the incompleteness of the dataset (2 data are still missing). The following data transmission (occurring 30 min later and represented by red dashed lines in Figure 4) completes the dataset (orange lines in Figure 4) and confirms the outcomes previously obtained with a level of confidence equal to 100%. Finally, the consequence of spike noise detection and removal is the time delay related to the acquisition of the complete centered dataset (1 h and 15 min for the example in Figure 4).

**Figure 5:**
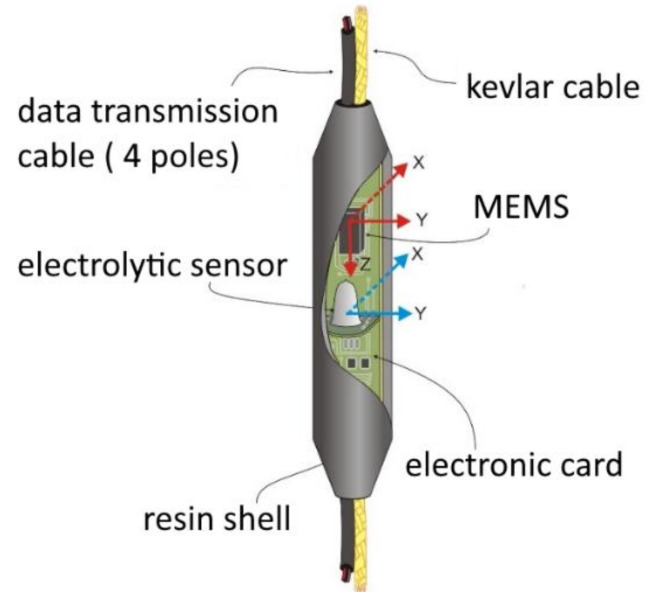
Tilt Link HR 3D sensor, equipped with 3D microelectromechanical system (MEMS) and 2D electrolytic cell, placed on the same electronic board, with instrumental axes aligned on the horizontal plane.

As stated before, multiparameter devices are one of the most relevant advantages of an innovative monitoring system. Similarly, it is possible to include different sensors measuring the same parameters in a single monitoring element in order to obtain a redundant measure and improve data reliability. Following this idea, the MUMS system permits the integration of two different tilt sensors at the same depth, giving redundancy to final outcomes. The double system (defined Tilt Link HR 3D) exploits a 3D MEMS and a 2D electrolytic cell (Figure 5), monitoring a wide range of differential displacements thanks to the high-resolution that characterizes the electrolytic cell and the 360-degree range of MEMS. In this configuration, results are obtained using the tilt value provided by the MEMS accelerometer, or the information of electro-level and the direction of movement recorded by the magnetometer. The instrumental axes of the two sensors coincide.

**Figure 6:**
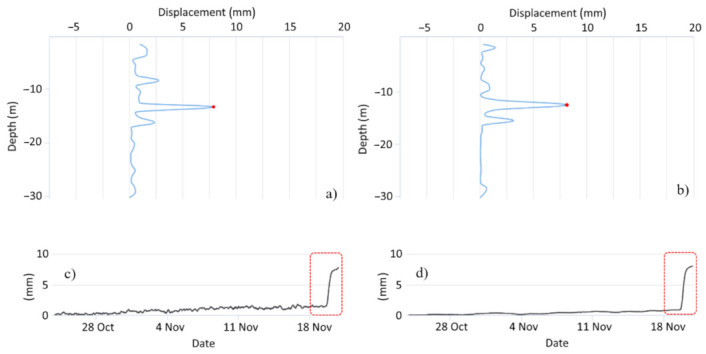
Comparison between local differential displacements recorded by (**a**) MEMS and (**b**) electrolytic cells along maximum grade direction and their evolution along time at a depth of 13 m (**c**,**d**), respectively).

Moreover, the redundancy measures provided by this approach play a major role in data analysis and validation processes [43]. Figure 6 shows a case history where the MUMS system was applied as an EWS to control a landslide threatening some houses. Specifically, the slope monitoring device included an automatic inclinometer (Vertical Array) integrating a total of 29 Tilt Link HR 3D sensors spaced 1 m one from each other and a piezometer to measure the water table level. Additionally, two tiltmeters and two crack meters were installed on the house walls to identify any effect induced by the slope movements on the buildings. In particular, the tiltmeters integrated a MEMS sensor and electrolytic tilt sensor, in a similar configuration to the one used in Tilt Link HR 3D. On the other hand, the two crack meters were analog devices featuring an mV/V output signal. All these tools were connected to a single control unit powered through the connection to the electrical line, while data were sent to the elaboration center via UMTS connection. On 19 November 2019, MEMS sensors recorded a rapid displacement of 6 mm localized at 13 m of depth that occurred over a time interval of 12 h. The comparison of local differential displacements recorded by MEMS (Figure 6a) and electrolytic cells (Figure 6b) along maximum grade direction gave a comfortable result, allowing the complete validation of the ongoing phenomenon. The evolution of displacement recorded at 13 m of depth over time highlights the improved accuracy and stability that distinguish electro-levels (Figure 6c) with respect to MEMS (Figure 6d) sensors, while the impulsive event was effectively recorded by both devices.

**Figure 7:**
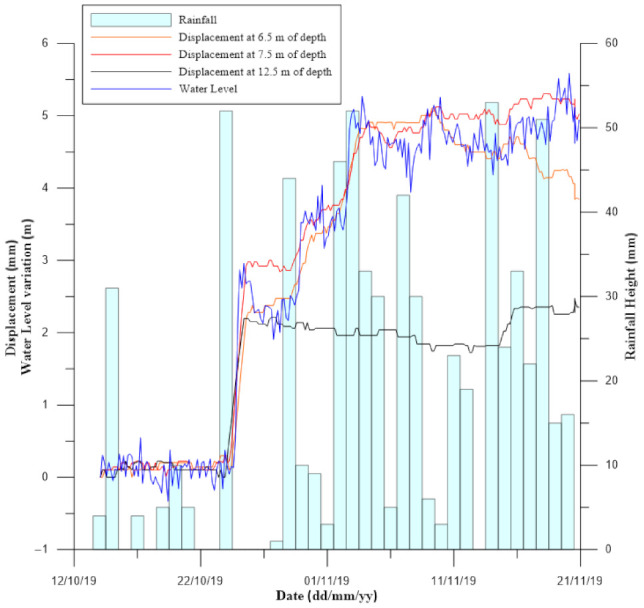
Comparison between rainfall height, water level variations and displacements recorded by a MUMS-based automatic inclinometer on a landslide in northern Italy.

As previously discussed, the management of a large number of multiparameter data with proper sampling frequencies could bring to the identification of significant cause-effects relationships, such as direct correlations between rainfalls, water levels and displacements over specific time periods. Figure 7 represents a case history in northern Italy where a MUMS-based automatic inclinometer was installed in order to monitor an active landslide that interacts with the construction site of a new road tunnel. Specifically, the monitoring tool is composed of 20 Tilt Link HR 3D V sensors, installed between 0.5 and 19.5 m of depth, and one piezometer. This sensor is designed to measure the absolute pore water pressure over time. Thanks to the presence of a barometer on-site, it is possible to remove the atmospheric component from the measure, thus obtaining the relative pore pressure. Then, the conversion of this value with previously computed calibration parameters allows for the evaluation of the water level variation over time. The instrumentation is still active and has monitored displacements, water level variations, and rainfall height, showing significant and interesting cause–effect correlations. In particular, the water level has been highly influenced by rainfalls, leading to a variation of 5 m in about two weeks and a stabilization that occurred later, during a period of heavy precipitations. Local differential displacements recorded on the maximum grade direction at a depth that ranges between 7.5 and 6.5 m show a strong correlation with the water level trend. It is also possible to observe the interaction of both water level and displacements, which tend to increase during the first significant impulsive events, reaching a situation that does not change even though the occurrence of continuous and relevant precipitations.

**Figure 8:**
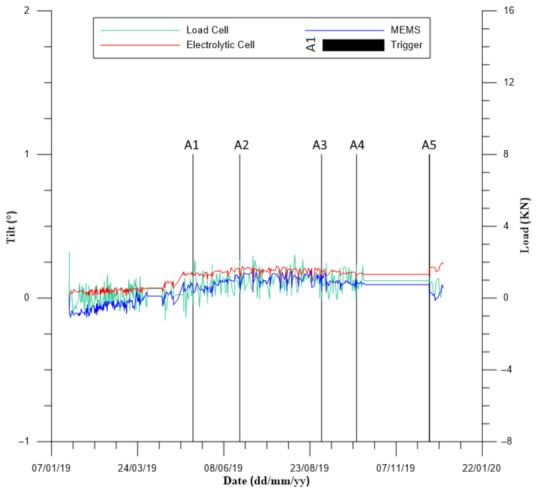
Trigger activations and related steel post tilt values recorded by MEMS and electro-level sensors, together with the mountain brace load identified by load cell sensor.

As mentioned in the previous paragraph, a trigger activation should lead to cross-check controls that analyze outcomes of related sensors. An application of this idea is displayed in Figure 8, showing a case history where a rockfall barrier is monitored through a rockfall safety network (RSN) MUMS system. RSN is a monitoring network composed of a main control unit radio connected with modules (defined BPM), each one equipped with a 3D MEMS, a 2D electrolytic cell and a load cell. Each single BPM module is installed directly on a different steel post of the barrier in order to monitor its rotation and inclination over time (thanks to MEMS and electro-level sensors) while the load cell controls the mountain brace load. The datalogger is powered by a lead–acid battery and a solar panel, while BPM modules exploit lithium-thionyl chloride batteries. A mechanical trigger connected to the control unit completes the monitoring system. When the trigger activates, the CU queries every device connected to the smart-mesh network and sends data to the elaboration center thanks to a UMTS connection. There, the software correlates the trigger to its related barrier, associating the BPM installed in correspondence with the potential impacts. The algorithm analyses the monitoring outcomes with a two-step procedure in order to check if the trigger activation corresponds to an actual event. In the first phase, the software defines a specific threshold for each sensor, established by a mean value computed from a dataset of monitoring outcomes recorded during previous days. If a predefined percentage of sensors overcomes these values, the algorithm activates the following step. At this stage, monitoring data are compared to reference parameters, as service energy level (SEL) or maximum energy level (MEL), that are defined by specific tests performed on the barrier. If the elaboration returns a positive result, then an alarm message is issued since the trigger is activated in correspondence with an exceptional event. On the other hand, no alarm is disseminated if one of these conditions is not verified since it means that the trigger sensors recorded a non-critical event. This procedure is intended to reduce the probability of issuing a false alarm, which can be identified if no variation is recorded by BPM modules at the trigger activation. An example of this occurrence is reported in Figure 8, where it is possible to observe how data trends recorded by MEMS, electro-level and load cell sensors do not show any variation despite several activations of the trigger installed on the barrier (activations are marked as A1 to A5 in Figure 8). In all these cases, the software automatically recognized the false alarm and did not disseminate any alert message. Further confirmation derived from on-site investigations, which verified the absence of material on the barrier after the trigger activations.

**Figure 9:**
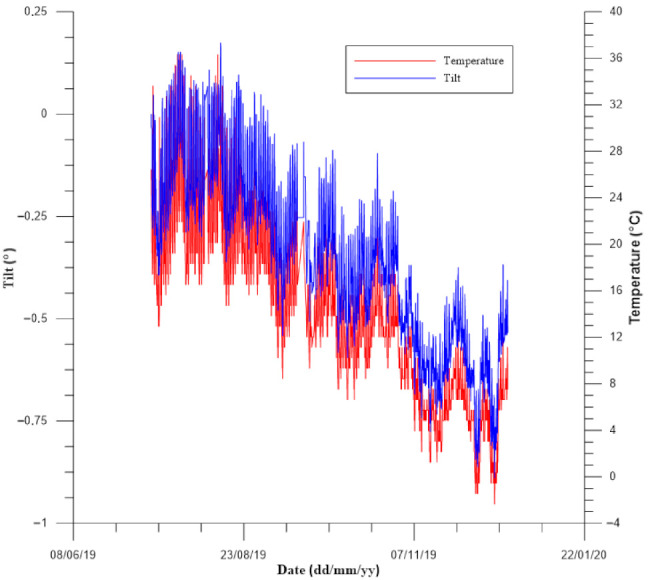
Comparison between tilt data and temperature recorded by MEMS sensor placed in a wall-mounted tiltmeter.

Another relevant advantage of the correlation between various physical entities is to provide more information in order to avoid interpretation errors and identify the phenomenon behavior. Figure 9 reports tilt data recorded by a tiltmeter placed on a building in order to identify possible landslide interactions with structural stability. The case study is the same previously described in Figure 5. It is possible to observe a trend, which could be interpreted as a slow and continuous tilt, related to a period of continuous rainfall. However, thanks to the thermometer integrated into the sensor, it was possible to observe a similar trend of temperature and tilt data. Therefore, thanks to the installation of two sensors, it was possible to find a relation between tilt variation and thermal effects, thus providing a more reliable monitoring data interpretation.

## 4. Conclusions

The enhancement of connection between different devices has been one of the most influential technological improvements that emerged in recent years. Thanks to Internet connectivity, it has been possible to improve the remote interaction of various technologies, thus creating the Internet-of-things (IoT) concept. As a result of their wide applicability and the several advantages deriving from its implementation, IoT principles have been integrated into a significant number of different processes and fields. In particular, the development of new automated and innovative monitoring tools has brought to the application of IoT to the geotechnical sector, leading to the design of advanced systems with the improved connection between different components.

One of the systems deriving from this new approach is the defined Internet of natural hazards (IoNH) and allows the remote collection and management of information that are fundamental for risk prevention purposes. As described in the present paper, the IoNH concept focuses on integrating IoT-based technologies to permit a constant check of instrumentation functionality, both in terms of sensor operativity and control unit conditions. Another advantage regards the possibility to control a large number of critical areas from a single control room thanks to completely automated procedures for data acquisition and elaboration. Moreover, the high sampling frequencies achievable by devices connected to the IoNH system give the possibility to perform statistical analyses to validate numerical results. Other features are the establishment of reliable cause–effect relationships related to multiparameter information, the activation of alert procedures with warning messages to the authority in charge of the direct activation of traffic lights and/or sirens. This paper presented a series of examples acquired from different case studies in order to underline the positive effects of the IoNH integration in a geotechnical monitoring system.

Finally, one of the most relevant topics related to automatic monitoring and early warning systems concerns risk communication strategies and procedures. In fact, when dealing with systems that can autonomously control the closure of roads, the preliminary evacuation of landslide areas, etc., it is necessary to implement specific actions that should be followed by the authority in charge and by the population. It is fundamental to keep in mind that technology should not be intended as a substitution of human decisions and evaluations, but it represents a valid tool for the proper and timely management of potentially hazardous situations.

## Data Availability

The data presented in this study are available on request from the corresponding author.
